# Primary and Booster COVID-19 Vaccination in Patients with Sjögren’s Disease: Data from the Longitudinal SAFER Cohort Study

**DOI:** 10.3390/vaccines13111152

**Published:** 2025-11-11

**Authors:** Maressa Barbosa Beloni Lirio, Ketty Lysie Libardi Lira Machado, Olindo Assis Martins-Filho, Samira Tatiyama Miyamoto, Yasmin Gurtler Pinheiro de Oliveira, Érica Vieira Serrano, José Geraldo Mill, Karina Rosemarie Lallemand Tapia, Lunara Baptista Ferreira, Juliana Ribeiro de Oliveira, Maria da Penha Gomes Gouvea, Laura Gonçalves Rodrigues Aguiar, Barbara Oliveira Souza, Vitor Alves Cruz, Ricardo Machado Xavier, Andréa Teixeira Carvalho, Viviane Angelina de Souza, Gilda Aparecida Ferreira, Odirlei André Monticielo, Edgard Torres dos Reis Neto, Emilia Inoue Sato, Gecilmara Salviato Pileggi, Valéria Valim

**Affiliations:** 1 Rheumatology Department, Cassiano Antônio Moraes University Hospital, Federal University of Espírito Santo (HUCAM-UFES/EBSERH), Vitória 29041-295, Espírito Santo, Brazilyasmingurtler@hotmail.com (Y.G.P.d.O.); mpgomesgov@gmail.com (M.d.P.G.G.);; 2 Integrated Group for Research in Biomarkers, René Rachou Institute, Oswaldo Cruz Foundation (FIOCRUZ-Minas), Belo Horizonte 30130-100, Minas Gerais, Brazil; 3 Faculty of Medicine, Federal University of Goiás (UFG), Goiânia 74690-900, Goiás, Brazil; 4 Porto Alegre Clinical Hospital, Federal University of Rio Grande do Sul (UFRGS), Porto Alegre 90010-150, Rio Grande do Sul, Brazil; 5 Faculty of Medicine, Federal University of Juiz de Fora, Juiz de Fora 36036-900, Minas Gerais, Brazil; vivi.reumato@gmail.com; 6 Locomotor System Department, Faculdade de Medicina, Universidade Federal de Minas Gerais (UFMG), Belo Horizonte 31270-901, Minas Gerais, Brazil; 7 Paulista School of Medicine (EPM), Federal University of São Paulo (UNIFESP), São Paulo 04023-062, São Paulo, Brazileisato@unifesp.br (E.I.S.);

**Keywords:** Sjögren’s Disease, COVID-19 vaccination, immunogenicity, safety, autoimmune diseases, booster dose

## Abstract

Introduction: The COVID-19 pandemic posed additional challenges for this vulnerable population, such as Sjögren’s disease (SjD), underscoring the need for effective and safe vaccination strategies. Objective: To evaluate the immunogenicity and safety of COVID-19 vaccines in patients with SjD. Methods: This prospective, observational, longitudinal study included SjD patients from the SAFER cohort. Immunogenicity was assessed via anti-spike IgG (IgG-S) titers using chemiluminescence reported as geometric mean titers (GMT) and fold increase in GMT (FI-GMT). Disease activity was evaluated using the ESSDAI score. Adverse events and COVID-19 infections were also monitored. Assessments were conducted at four time points: pre-first dose (T1), pre-second dose (T2), pre-booster (T3), and four weeks post-booster (T4). Primary vaccination involved ChAdOx1 nCoV-19 or inactivated vaccine (CoronaVac), and boosters were either homologous (ChAdOx1 nCoV-19) or heterologous (BNT162b2). Results: Among 51 participants (mean age 46 years; 90% female), 41% had comorbidities and 27% (n = 14/51) were highly immunosuppressed. Among those 73% (n = 37/51) under low immunosuppression, n = 8/51 (13%) were not using any medication. At baseline, 11% (n = 4/35) showed moderate/high disease activity, which decreased to 6.5% (n = 2/31) at T4. Primary vaccination was ChAdOx1 in 94% (n = 48/51) and CoronaVac in 6% (n = 3/51); 73% (n = 37/51) received heterologous and 27% (n = 14/51) homologous boosters. COVID-19 infection post-booster occurred in 20% (n = 10/51). Seroconversion rates reached nearly 100% across all medication subgroups except for biologic users, who showed delayed but stable seroconversion by T4. IgG-S titers increased progressively through T4. Primary immunization induced an ascending GMT in both vaccine types. At T4, the GMT was significantly higher in the BNT162b2 group (2148.03 [1452.05–3155.84]; *p* < 0.001; 95% CI) than in the ChAdOx1 group (324.29 [107.92–974.48]; *p* < 0.001; 95% CI); the fold-increase in immune response was six times greater with BNT162b2 (5.98 [2.97–12.03]; *p* = 0.001; 95% CI). Seroconversion was 100% in the heterologous group versus 83% in the homologous group (*p* > 0.01). Those with prior infection showed significantly higher titers, particularly at T2 and T3 (*p* < 0.001 for T1–T3). Adverse events were mild and not statistically significant. Multivariate regression confirmed BNT162b2 as an independent factor for higher antibody titers. Conclusion: COVID-19 vaccination in patients with SjD was safe and induced high anti-spike antibody titers and seropositivity. Heterologous boosting, particularly with BNT162b2, demonstrated superior immunogenicity. No association was found between vaccination and SjD disease flares or worsening activity.

## 1. Introduction

Coronavirus disease (COVID-19) is an infectious illness caused by the SARS-CoV-2 virus, first identified in Wuhan, China, in December 2019. Its clinical presentation ranges from asymptomatic to severe forms, including acute respiratory distress syndrome and death. The pandemic has caused millions of deaths worldwide and has been associated with an increased risk of complications in individuals with comorbidities, especially those who are immunosuppressed [1,2].

Vaccination is an effective preventive measure to reduce morbidity and mortality from infectious diseases and is particularly important for immunosuppressed populations, such as patients with Sjögren’s disease (SjD) [3,4].

The SjD is a systemic autoimmune condition marked by lymphoplasmacytic infiltration of exocrine glands, resulting in glandular and extraglandular dysfunction. It affects mainly women, especially in their 40s and 50s, with a prevalence around 0.2% [5,6]. SjD is a heterogeneous disease, with phenotypes that vary in progression and prognosis, suggesting distinct underlying pathophysiological mechanisms. Systemic treatment often involves glucocorticoids, synthetic DMARDs, immunosuppressants, and biologics, which may impair protective immune responses [6,7,8].

Some studies have shown that COVID-19 vaccine effectiveness in people with immune-mediated inflammatory diseases varies depending on the type and severity of the underlying condition, the degree of immunosuppression (e.g., the use of synthetic or biological disease-modifying antirheumatic drugs), the presence of multimorbidity, and the vaccine regimens used [9,10,11,12,13].

In this context, only three studies to date have evaluated vaccine responses following primary immunization with one or two doses using different vaccine platforms, in SjD [9,10,11]. These studies reported lower immune responses in patients compared to controls [9,10]. The safety profile was considered acceptable, with no serious adverse events reported, although some patients experienced worsening of sicca symptoms or disease flares [11].

No studies have yet evaluated the immune response following a booster dose in patients with SjD. This study was designed to assess the immunogenicity and safety of COVID-19 vaccines in this population, including both primary and booster regimens, comparing homologous and heterologous strategies in a prospective cohort of patients with SjD.

In this study, participants received vaccines based on different technological platforms. These included inactivated whole-virus vaccines, which use chemically inactivated SARS-CoV-2 particles to induce an immune response; viral vector vaccines, which employ a non-replicating adenovirus to deliver the genetic sequence encoding the spike protein; and messenger RNA (mRNA) vaccines, which directly provide the genetic instructions for host cells to produce the spike protein and elicit both humoral and cellular immune responses. This diversity of platforms reflects the vaccination strategies available in Brazil during the study period and provides an opportunity to assess immune responses across distinct vaccine technologies in patients with SjD [14].

## 2. Materials and Methods

This is an observational, longitudinal, prospective, not controlled study designed to evaluate patients with SjD vaccinated with either a non-replicating viral vector vaccine (ChAdOx1 nCoV-19, AstraZeneca, Oxford, UK) or an inactivated adsorbed vaccine (CoronaVac, Sinovac Biotech, Beijing, China) as the primary dose, and messenger RNA vaccine (BNT162b2, Pfizer-BioNTech, Mainz, Germany) or ChAdOx1 nCoV-19 as a booster dose.

This study is an analysis of data collected from the SAFER study (Safety, Efficacy, and Duration of Immunity after SARS-CoV-2 Vaccination in Patients with Chronic Immune-Mediated Inflammatory Diseases), a prospective, multicenter, phase IV real-world study sponsored by the Brazilian Society of Rheumatology (SBR) and the Department of Science and Technology of the Brazilian Ministry of Health (DECIT-MS) [12].

### 2.1. Study Population

Participants were recruited from referral centers at university hospitals in three different states (Espírito Santo, Goiás, and São Paulo) across two Brazilian regions (Central-West and Southeast).

### 2.2. Inclusion Criteria

All participants included had a diagnosis of SjD according to the 2016 EULAR/ACR international classification criteria [15], were over 18 years old, able to understand and read the Informed Consent Form, and had medical indication and authorization for vaccination. Only those who completed the COVID-19 vaccination schedule with three doses and attended all four scheduled blood collections—from baseline (pre-vaccination) to 28 days after the third dose administered—were included in the analyses [12].

At all visits, information regarding the date, site of administration, vaccine type, vaccination schedule, and adverse events was recorded [13].

### 2.3. Exclusion Criteria

Excluded were individuals with other causes of immunosuppression—such as HIV, organ transplantation, primary immunodeficiency, malignancies, prior history of thymic disease, or use of rituximab in the past six months—as well as pregnant women, individuals with a history of severe adverse reactions to any previously administered vaccine, recipients of vaccine platforms other than inactivated vaccines, ChAdOx1 nCoV-19, or BNT162b2, those with incomplete immunization schedules, and those who missed any subsequent blood collections.

### 2.4. Vaccination Schedules

Vaccination was performed using homologous schedules with three doses of ChAdOx1 nCoV-19 (AstraZeneca, Oxford, UK), and heterologous schedules with two doses of inactivated adsorbed vaccine (CoronaVac, Sinovac Biotech) followed by a booster dose of BNT162b2 (Pfizer-BioNTech), or two doses of ChAdOx1 nCoV-19 (AstraZeneca, Oxford, UK) followed by a BNT162b2 (Pfizer-BioNTech) booster.

### 2.5. Ethical Procedures

The study was approved by the National Research Ethics Committee (CONEP) under protocol (CAAE: 43479221.0.1001.5505), on 30 April 2021, and conducted in accordance with Good Clinical Practice guidelines (GCP), the International Council for Harmonization (ICH), the ethical principles of the Declaration of Helsinki, Brazilian legislation (Resolution 466/2012), and ethics committee guidelines.

All participants signed the Informed Consent Form before data collection began. Participation was voluntary and unpaid. However, they received reimbursements for expenses related to food and transportation, as provided by local ethical standards.

To ensure confidentiality, personal identification data were anonymized and protected according to national and international regulations. Only medical researchers and nurses from the research team had access to participants’ medical records, solely for data retrieval purposes.

### 2.6. Clinical Data

Sociodemographic data, presence of comorbidities, disease characteristics and severity, treatments, clinical features, and SARS-CoV-2 infection outcomes (prior or acquired during the study) were recorded. Disease activity was assessed by the EULAR Sjögren’s Syndrome Disease Activity Index (ESSDAI) [16].

The degree of immunosuppression was defined according to the medication regimen, including drug type, dose, and route of administration [12,17].

Patients considered non-immunosuppressed included those not using any pharmacological treatment, as well as those using drugs with mild or no immunomodulatory effect, such as exclusive use of sulfasalazine, hydroxychloroquine, or mesalazine, topical corticosteroids, inhaled corticosteroids, or locally administered corticosteroids (periarticular or intra-articular) [12,17].

Low immunosuppression was attributed to the use of methotrexate up to 20 mg/week, leflunomide up to 20 mg/day, and systemic corticosteroids at doses up to 20 mg/day of prednisone or equivalent. High immunosuppression included prolonged corticosteroid use at doses ≥20 mg/day of prednisone for 14 days or more, pulse therapy with methylprednisolone, use of mycophenolate mofetil or sodium, cyclosporine, cyclophosphamide, tacrolimus, azathioprine, JAK inhibitors (such as tofacitinib), and biologic disease-modifying antirheumatic drugs (b-DMARDs), such as TNF, IL-6, IL-17 blockers, co-stimulation inhibitors, and anti-CD20 agents [12,17].

### 2.7. Immunogenicity Assessment

Humoral immunogenicity was evaluated at four time points: pre-first dose (T1), pre-second dose (T2), pre-booster (T3), and four weeks post-booster (T4). The humoral immune response was analyzed by measuring IgG antibodies specific for the receptor-binding domain (RBD) of the SARS-CoV-2 Spike protein (IgG-S), using the Architect SARS-CoV-2 Quanti II chemiluminescence assay (SARS-CoV-2 IgG II Quant assay, Abbott). Obtained values were transformed using the natural logarithm function (ln), allowing standardized analysis of data as “ln-transformed IgG.” Based on this transformation, the geometric mean titers (GMT) and geometric mean fold increase (FI-GMT) were calculated to compare immune responses between groups and vaccination times. Results were expressed in BAU/mL (Binding Antibody Units per milliliter), following WHO standardization. Conversion from AU/mL to BAU/mL was based on the WHO international standard 20/136, using a manufacturer-provided conversion factor (e.g., for Abbott, 1 AU/mL = 0.142 BAU/mL, when applicable). The cut-off value of 50 AU/mL or 7.1 BAU/mL indicates positivity for IgG antibodies against the Spike protein (S).

### 2.8. Vaccine Effectiveness Assessment

Vaccine effectiveness was analyzed based on the occurrence of positive COVID-19 cases 15 days after the first, second, and third doses at each evaluation time, considering different vaccines used, primary scheme, and booster.

For COVID-19 case analysis, new cases were defined as those confirmed by RT-PCR, and reinfection was defined as occurring at least 180 days after a prior diagnosis.

Infection occurrence was analyzed considering the primary schedule, defined as the period from 15 days after the second dose until the booster dose administration date. After the booster dose, the interval considered was from 15 days after the booster dose until the fourth dose date.

Prior viral exposure was assessed by IgG-S levels ≥ 7.1 BAU/mL before the first vaccine dose (T1).

### 2.9. Safety

Adverse events were monitored by recording local symptoms (e.g., redness, warmth, swelling, local pain) and systemic symptoms (fever, headache, new or worsened myalgia, new or worsened arthralgia, fatigue, nausea, vomiting, diarrhea, pruritus, erythema) reported by participants through a structured diary during the first 28 days after vaccination (reactogenicity), and active search by the evaluator at all assessment times. This analysis was stratified by vaccine type.

Disease activity was monitored by ESSDAI at T1 (baseline) and T4 (28 days after booster dose).

### 2.10. Statistical Analysis

Data were tabulated and analyzed using Stata Statistical Software: Release 17. College Station, TX: StataCorp LLC; 2021. Proportions between groups were compared using chi-square or Fisher’s exact test, as appropriate, for categorical variables. For continuous variables, mean and standard deviation, as well as median and interquartile range, were calculated, and comparisons were performed using ANOVA, Kruskal–Wallis, or Wilcoxon/Mann–Whitney tests, depending on distribution. Disease activity before and after vaccination was assessed with McNemar’s test.

To address the potential impact of the relatively small sample size and multiple comparisons, non-parametric methods were applied when appropriate, and Bonferroni correction was used to reduce the risk of type I error. Results are reported with 95% confidence intervals, and the imprecision associated with the limited sample size was explicitly acknowledged in the interpretation of findings.

Data analysis focused on six prespecified outcomes encompassing vaccine immunogenicity, effectiveness, and safety, disease activity, as well as potential sociodemographic and clinical determinants of immune response.

(1) Humoral immunogenicity was evaluated using ln-transformed IgG titers and geometric mean fold increase (FI-GMT); group and time comparisons were conducted with Wilcoxon/Mann–Whitney non-parametric tests, an approach suited to small sample sizes and non-normal distributions. Bonferroni correction was applied in the presence of multiple paired comparisons.
(2) Seroconversion (SC) was summarized descriptively as the proportion of previously seronegative participants who converted in each group.
(3) Predictors of IgG at T4 were explored by simple linear regression, with biologically relevant factors subsequently included in multiple regression models.
(4) Vaccine effectiveness, defined as RT-PCR–confirmed COVID-19 cases, and
(5) Safety, based on local and systemic post-vaccination symptoms, was analyzed with Fisher’s exact test to account for small cell counts.
(6) Disease activity was described using ESSDAI and physician global assessment, adjusting for baseline activity.

All analyses were performed using R version 4.2 (R Core Team, 2023) and RStudio version 2024.9.1 (RStudio: Integrated Development Environment for R. Posit Software, PBC, Boston, MA, USA. Available at: http://www.posit.co/) with the tidyverse, gtsummary, and rstatix packages. A two-sided significance level of 5% and 95% confidence intervals were used.

## 3. Results

**Figure 1:**
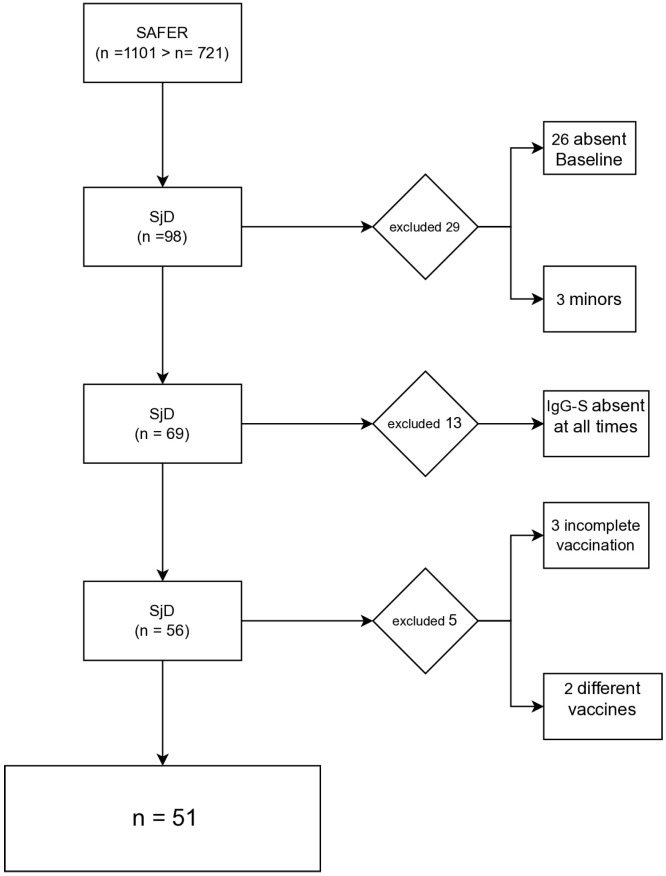
Diagram of selection of participants with Sjögren’s Disease (SD) included in the SAFER cohort.

A total of 98 participants with SjD were found in the SAFER database via RedCap. Of these, 26 were excluded due to missing baseline data, and 3 were adolescents. Subsequently, 13 participants were excluded for lacking IgG-S results at all time points. Additionally, 3 participants with incomplete vaccination schedules and 2 with non-standard vaccine combinations (CoronaVac/ChAdOx1 nCoV-19 for T1/T2, BNT162b2/ChAdOx1 nCoV-19 for T3) were excluded. In total, 51 participants were included in the analysis (Figure 1).

**Figure 2:**
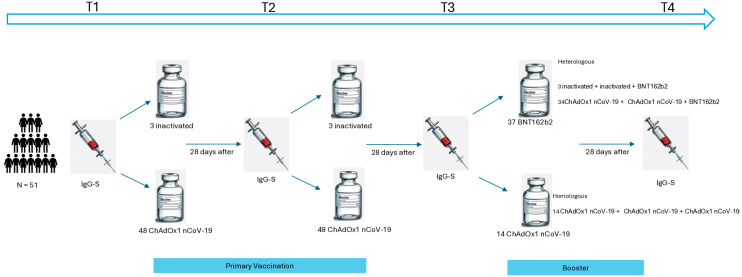
Study design of primary and booster vaccination against COVID-19 in primary Sjögren’s disease.

Data were collected from participants who received a primary vaccination regimen with CoronaVac or ChAdOx1 nCoV-19, and a booster dose with either ChAdOx1 nCoV-19 or BNT162b2. Participants were assessed at four time points: before the first dose (T1), before the second dose (T2), before the third dose (T3), and four weeks after the third dose (T4) (Figure 2).

Demographic and clinical characteristics of the study population are detailed in Table 1. The mean age was 46 ± 12 years, with a predominance of females (n = 46, 90%). Most participants self-identified as mixed race (n = 29, 57%), and nearly half reported comorbidities (n = 21, 41%). The median BMI was 27 kg/m^2^ (IQR: 25–31). Among those tested, 94% (33/35) had a positive antinuclear factor (ANA), and 57% (25/44) were positive for anti-Ro/SSA antibodies.

In terms of immunosuppressive status, 27% (n = 14/51) were under high immunosuppression. Among those 73% (n = 37/51) under low immunosuppression, n = 8/51 (16%) were not using any medication. Regarding medication, 65% (n = 33) were on hydroxychloroquine, 14% (n = 7) on methotrexate, 5.9% (n = 3) on leflunomide, 14% (n = 7) on azathioprine, 3.9% (n = 2) on mycophenolate, 5.9% (n = 3) on oral corticosteroids, and 5.9% (n = 5) on biologics—four on abatacept and one on tocilizumab. At baseline, 89% (31/35) were in remission or had low disease activity.

### 3.1. Vaccination Regimens

Table 2 details the COVID-19 vaccine regimens received. The primary immunization series consisted of two doses of ChAdOx1 nCoV-19 in 94% (n = 48) and CoronaVac in 5.9% (n = 3). The third dose was BNT162b2 in 73% (n = 37) or ChAdOx1 nCoV-19 in 27% (n = 14).

Regarding vaccine regimen type, 73% (n = 37) received a heterologous scheme (different vaccines), and 27% (n = 14) received a homologous scheme (same vaccine). Full regimen distribution:

ChAdOx1 + ChAdOx1 + BNT162b2: 67% (n = 34)

ChAdOx1 + ChAdOx1 + ChAdOx1: 27% (n = 14)

CoronaVac + CoronaVac + BNT162b2: 5.9% (n = 3)

Inter-dose intervals followed Brazilian Ministry of Health guidelines: 30 days for inactivated vaccines and 60 days for ChAdOx1. The third dose was administered approximately two months after the second. Among those who received inactivated vaccines (n = 3), the mean interval between first and second doses was 32 ± 8 days, and between the second and third, 124 ± 42 days. For those vaccinated with ChAdOx1 (n = 48), the mean intervals were 78 ± 3 days (first to second) and 59 ± 13 days (second to third).

### 3.2. Seroconversion Rates by Medication

**Figure 3:**
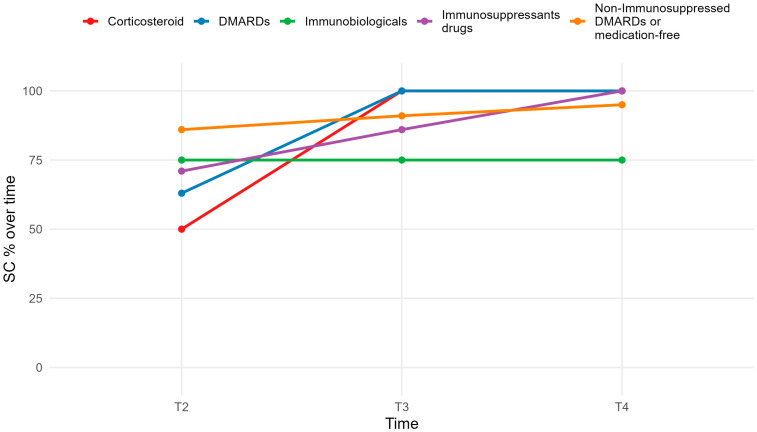
Seroconversion rate in medication use.

As shown in Figure 3, at T3, all medication categories had seroconversion rates above 80%, with 100% among those on conventional DMARDs or corticosteroids (*p* > 0.01). Only biologic users had rates below 80%, which remained consistent across time points.

### 3.3. Humoral Immunogenicity Evaluation

**Figure 4:**
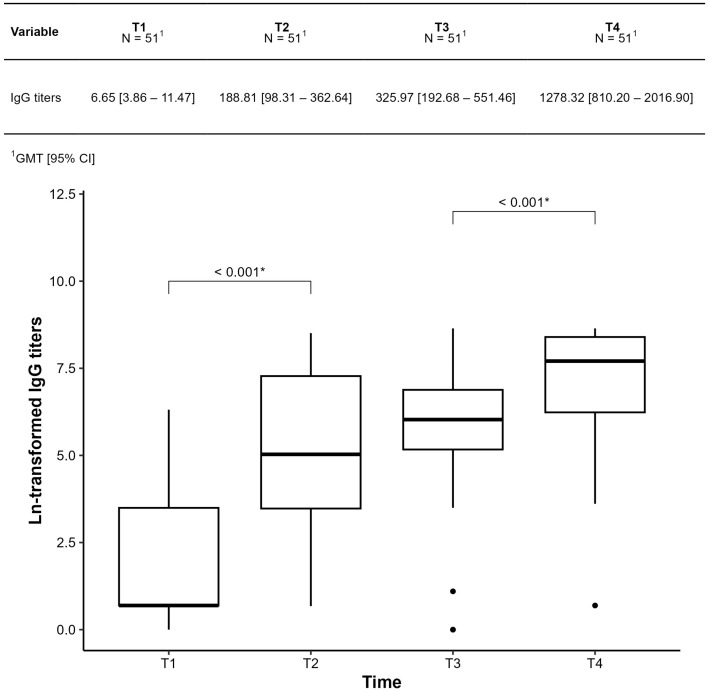
Comparison of IgG-S titers at each time point throughout the period was collected in patients with Sjogren’s disease at baseline (T1); 28 days after the first dose (T2); 28 days after the second dose (T3); 28 days after the third dose (T4). The results were presented as geometric mean IgG titers (GMT). * Statistically significant *p* < 0.001.

IgG-S titers were assessed at four time points. Geometric mean titers (GMTs) and their confidence intervals are shown in Figure 4. IgG-S levels increased significantly between T1–T2 and T3–T4, but not between T2–T3 (*p* = 0.124).

**Figure 5:**
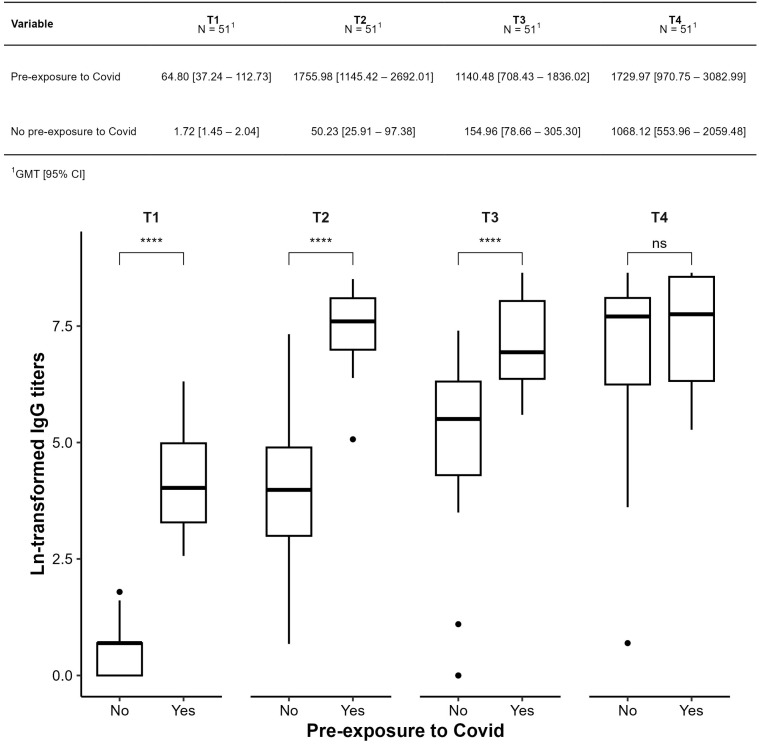
Comparison of geometric means of IgG anti-spike antibody titers against SARS-CoV-2 (IgG-S) between subgroups with (yes) and without prior exposure (no) to COVID-19, before the start of the study. **** *p* ≤ 0.0001; ns: not significant.

Figure 5 compares participants with and without prior COVID-19 exposure. Those with prior infection showed significantly higher titers, particularly at T2 and T3 (*p* < 0.001 for T1–T3). In the unexposed group, all pairwise time comparisons were significant (*p* < 0.001 or *p* = 0.002), suggesting a robust vaccine response.

**Figure 6:**
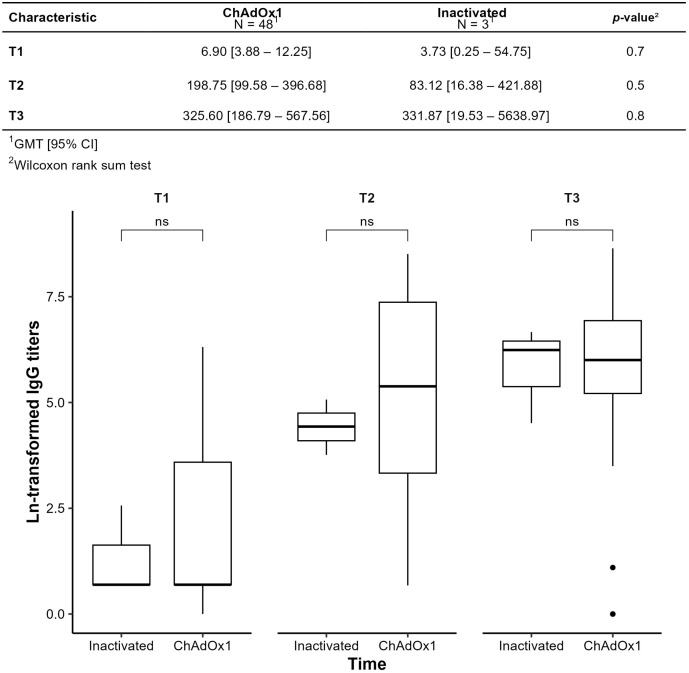
Humoral immunogenicity of primary immunization observed by comparing the geometric means of SARS-CoV-2 anti-spike antibody titers (IgG-S) over time; ns: not significant.

Figure 6 compares GMTs between primary vaccination with inactivated vaccine and ChAdOx1. In the ChAdOx1 group, GMTs increased significantly from T1 to T2 (*p* < 0.001), but not from T2 to T3 (*p* = 0.2). The small, inactivated vaccine subgroup limited between-group comparisons, with no significant differences at any time point (*p* > 0.05).

**Figure 7:**
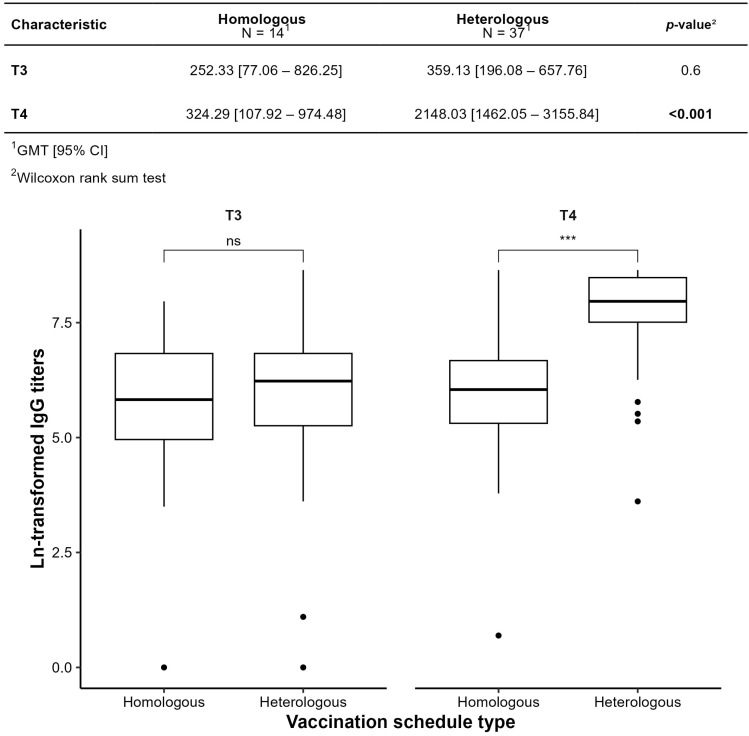
Comparison by type of vaccination schedule Homologous and Heterologous by geometric mean titer (GMT). *** *p* ≤ 0.001; ns: not significant.

Figure 7 compares GMTs between homologous (ChAdOx1-only) and heterologous (inactivated vaccine or ChAdOx1 + BNT162b2) regimens. At T4, heterologous regimens yielded significantly higher GMTs (2148.03 vs. 324.29, *p* < 0.001). In the homologous group, the T3–T4 increase was not statistically significant (*p* = 0.502; only 30% increase), suggesting limited boost efficacy. In the heterologous group, the increase was significant (*p* < 0.001), with a sixfold rise (5.98 [2.87–12.03]). Seroconversion occurred in 83% (homologous) vs. 100% (heterologous).

At T3, GMTs were similar across groups. At T4, the inactivated vaccine + inactivated vaccine + BNT162b2 group had the highest GMT (2962.85), followed by ChAdOx1 + ChAdOx1 + BNT162b2 (2087.93) and ChAdOx1-only (324.29). This suggests that heterologous regimens with BNT162b2 induced stronger antibody responses. There was no significant difference between inactivated vaccine-based and ChAdOx1-based heterologous regimens (*p* = 1), indicating that heterologous boosting with BNT162b2 was more effective regardless of primary vaccine type.

Univariate regression (Table 3) assessed clinical and demographic variables associated with humoral response at T4. Variables such as sex, age, comorbidities, prior COVID-19, DMARD/immunosuppressant use, and primary vaccine type were not significantly associated with IgG levels at T4 (*p* > 0.05). The only statistically significant predictor was the booster with BNT162b2, which led to higher IgG levels compared to ChAdOx1 (*p* < 0.001). Biologic use showed a trend toward lower titers (−1.3), but this was not statistically significant (*p* = 0.087). In multivariate regression, BNT162b2 remained the only independent predictor of higher antibody titers.

### 3.4. Vaccine Effectiveness

Two new COVID-19 cases (3.9%) occurred during primary vaccination (between the 1st and 2nd doses), and one case (2%) occurred between the second dose. After the booster, 20% (n = 10) became infected. The post-booster observation period was variable and included waves of outbreaks and new variants.

### 3.5. Vaccine Safety

Safety was assessed based on self-reported adverse events, categorized by type (local/systemic), specific symptoms, and duration (Table 4, Table 5 and Table 6).

For primary vaccination, local reactions occurred in 100% of inactivated vaccine recipients (n = 3/3) and in 92% of ChAdOx1 recipients (n = 44/48), with no statistical difference (*p* > 0.9). Systemic reactions were significantly more common with ChAdOx1 (92%) compared to inactivated vaccines (33%) (*p* = 0.033). Symptoms like headache, myalgia, arthralgia, fever, and nausea were more frequent and lasted longer with ChAdOx1. Skin lesions and joint pain were also more persistent in the ChAdOx1 group.

For the booster dose, both ChAdOx1 and BNT162b2 showed similar safety profiles: local/systemic reaction rates were 71% (n = 10/14) and 82% (n = 27/33), respectively (*p* = 0.5). Common symptoms included local pain, headache, fatigue, myalgia, and arthralgia, with frequencies ranging from 50% to 79%.

Symptom duration was also comparable. Most events are resolved within 1–5 days. Some isolated cases of nausea and joint pain lasted up to 28 days in both groups, with no significant differences.

## 4. Discussion

This prospective, longitudinal observational study systematically evaluated the immunogenicity, safety, and clinical impact of SARS-CoV-2 vaccination in patients with SjD, covering both primary and booster regimens and comparing homologous and heterologous schedules. The findings demonstrate that vaccination was highly immunogenic and generally safe in this population, with no evidence of increased disease activity or severe adverse events attributable to vaccination.

Our results show a robust seroconversion rate (90.5%) after the primary vaccination scheme, which is consistent with findings in other autoimmune rheumatic disease (ARD) populations [12,18,19,20,21,22,23,24,25,26] and other studies in SjD [9,10,11]. The booster dose further enhanced the immune response, achieving a 97.6% seroconversion rate. Importantly, the geometric mean titers (GMT) of anti-S IgG antibodies significantly increased post-booster and remained elevated over time. These findings highlight the booster dose as essential for sustaining an adequate humoral response in SjD patients, which is particularly relevant given emerging data on waning immunity and new variants of concern [12,13,20,21,22,23,24,25,26].

The serological response we observed is comparable to that reported by Pasoto et al. (2022), who found seroconversion rates above 90% in Brazilian patients with SjD after two doses of inactivated vaccine [9]. However, our study extends these findings by including patients vaccinated with a variety of platforms, providing real-world data on vaccine effectiveness across different regimens. Additionally, unlike prior studies, we provide longitudinal follow-up of antibody kinetics and capture the effect of heterologous and homologous booster regimens [13].

Disease activity, measured using the ESSDAI, remained stable throughout the study period. This suggests that both primary and booster vaccinations are safe in patients with SjD, as they do not lead to clinically relevant increases in systemic disease activity. These findings are aligned with studies in other systemic autoimmune diseases, such as systemic lupus erythematosus and rheumatoid arthritis [12,27,28], and are of particular importance for clinicians who may be concerned about potential disease flares following immunization.

Most patients in our cohort presented with low or inactive disease activity, which reflects the typical distribution observed in clinical practice and in previous studies of SjD. We acknowledge that this profile limits the ability to fully evaluate vaccine safety in the context of high disease activity. Nevertheless, current evidence indicates that the vaccine platforms assessed in our study are considered safe for patients with autoimmune diseases, regardless of disease activity status. Therefore, while our findings mainly apply to patients with mild disease, they remain consistent with the broader literature supporting the safety of COVID-19 vaccination in systemic autoimmune conditions.

Data from the Sjögren Big Data Consortium involving 1237 SjD patients revealed a high rate of humoral response, with seropositivity in approximately 95% of individuals after the first vaccine dose and a relatively low incidence (11%) of disease flare-ups [11]. Our study reported no post-vaccination exacerbations. On the contrary, a reduction in disease activity was observed, likely due to the smaller sample size, low baseline disease activity, and potential differences in therapeutic regimens. Previous studies have shown that vaccination rarely induces disease flares, and when it does, these tend to be mild and not causally established [9,10,11,12].

The rate of COVID-19 breakthrough infections observed in our cohort was consistent with rates reported in the general population during the Omicron wave in Brazil [29]. Approximately 20% of breakthrough infections occurred post-booster, likely influenced by emerging variants or waning immunity. Importantly, most infections were mild and did not require hospitalization. These findings underscore the role of humoral immunity in preventing symptomatic diseases and reinforce the importance of booster vaccinations for high-risk populations, including those with autoimmune diseases [30,31].

Due to the small sample size, robust comparisons between primary regimens were limited. Nevertheless, a trend toward stronger initial immunogenicity with ChAdOx1 was noted, consistent with existing literature on viral vector vaccines, which often elicit a more potent early immune response. In contrast, the inactivated vaccine group displayed a more stable yet potentially less intense response over time.

Notably, patients receiving high levels of immunosuppression exhibited reduced seroconversion rates compared to those with mild or no immunosuppression, with rates remaining below 80% throughout follow-up. This suggests the need for more stringent monitoring and potentially additional booster doses in this subgroup.

Booster regimens incorporating BNT162b2 demonstrated markedly superior immunogenicity compared to homologous ChAdOx1 regimens, highlighting the critical role of mRNA boosters regardless of the primary regimen. These results align with current literature demonstrating the superior immunogenicity of mRNA vaccines, especially when used heterologous, supporting their preferential use in immunocompromised or immune-dysregulated populations such as patients with SjD [12,19,21,24,25]. Multivariate analysis identified BNT162b2 booster administration as the only independent predictor of higher anti-spike IgG titers at T4 (*p* < 0.001), while variables such as age, comorbidities, prior infection, sex, and initial vaccine type (inactivated vaccine or ChAdOx1) showed no significant associations.

In our cohort, a small subset of patients was receiving biologic agents, a group in whom reduced immunogenicity was observed. This finding is consistent with evidence from other immune-mediated inflammatory diseases, where biologic or targeted immunosuppressive therapies have been shown to impair vaccine-induced immune responses. Importantly, recent data also indicate that COVID-19 vaccination remains safe in pharmacologically immunosuppressed patients with autoimmune conditions, without an increased risk of severe adverse events [22]. These results reinforce that, although immunosuppressive therapy may attenuate antibody titers, vaccination should still be strongly recommended in this population, given its favorable safety profile and the protection conferred against severe COVID-19 outcomes.

The incidence of serious adverse events (SAEs) was low, with no vaccine-related deaths reported, supporting the overall safety of SARS-CoV-2 vaccines in patients with SjD, including those under immunosuppressive treatment. Only one SAE—neuromyelitis optica following a BNT162b2 booster—was considered potentially related to vaccination. Nonetheless, the safety profile was largely favorable, characterized predominantly by mild and self-limiting adverse events (AEs), consistent with those reported in large-scale immunization programs [19].

Local reactions were common across all vaccine platforms, observed in 100% of participants receiving inactivated virus platform and 92% of those receiving ChAdOx1 during the primary series, and in 71% ChAdOx1 and 82% BNT162b2 during the booster phase. These findings are in line with previous reports indicating that injection site pain is the most frequent local reaction and is typically transient [9,10,11,27,28,31].

Systemic reactogenicity was significantly more frequent with ChAdOx1 during the primary vaccination (92%) compared to inactivated vaccine (33%) (*p* = 0.033), which aligns with existing data showing a higher incidence of systemic symptoms associated with viral vector vaccines [11]. Common ChAdOx1-related systemic symptoms included fatigue (73%), headache (73%), and arthralgia (73%), forming a characteristic short-term inflammatory profile. For the booster dose, the frequency of systemic reactions was similar between BNT162b2 and ChAdOx, though pruritus was more frequently reported with BNT162b2 (67%).

Analysis of symptom duration revealed that most AEs were short-lived, with no significant differences across vaccine types. Arthralgia emerged as the most prolonged symptom, persisting for up to 28 days. Nausea and vomiting had a longer median duration in ChAdOx1 recipients (15 days) compared to those who received BNT162b2 (5 days), although this difference was not statistically significant, potentially reflecting a more pronounced systemic inflammatory response elicited by vector-based vaccines [11,27,28]. Importantly, despite the higher frequency of systemic AEs in ChAdOx1 recipients, no severe adverse events were directly attributed to any vaccine platform, reinforcing the safety of both inactivated virus and viral vector vaccines in immunocompromised populations.

Importantly, additional analyses including patients excluded for incomplete follow-up yielded results consistent with those presented here, further reinforcing the robustness of our findings.

This study has several strengths, including prospective design, use of standardized instruments to assess disease activity, and centralized serological testing. However, some limitations must be acknowledged. First, the sample size is relatively small and may not fully capture the heterogeneity of SjD. Second, T-cell-mediated immunity was not evaluated, which could provide complementary insights into vaccine-induced protection [32]. These analyses were planned, and T-cell response data were collected in parallel. They are currently under evaluation and will be presented in a forthcoming manuscript. Finally, some patients were under immunosuppressive treatment, which may affect antibody responses and generalizability [8].

## 5. Conclusions

In conclusion, full COVID-19 vaccination was safe and induced high anti-spike antibody titers in patients with SjD after short follow-up. The booster dose significantly enhances and sustains humoral immune responses, without exacerbating systemic disease activity. These findings support the continued recommendation for booster doses in this population.

## Figures and Tables

**Table 1:** Demographic and clinical characteristics of the population with Sjögren’s disease.

Characteristic	N = 51 ^1^	Characteristic	N = 51 ^1^
Age	45.86 (±11.72)	Diagnosis time (In years)	7.0 [4.0–11.0]
Gender		Immunosuppression level	
Female	46/51 (90%)	No/Low	37/51 (73%)
Race		High	14/51 (27%)
Brown	29/51 (57%)	Medication	
White	21/51 (41%)	Immunobiologicals	5/51 (9.8%)
Black	1/51 (2.0%)	Immunosuppressive Drugs	9/51 (18%)
Comorbidities	21/51 (41%)	Corticosteroid	3/51 (18%)
BMI—Categorical		DMARDs	10/51 (20%)
1—Underweight (BMI < 18.5)	1/51 (2.0%)	HCQ	33/51 (65%)
2—Healthy Weight (BMI 18.5–24.9)	11/51 (22%)	Free	8/51 (16%)
3—Overweight (BMI 25–29.9)	23/51 (45%)	ESSDAI	
4—Obesity (BMI > 30)	16/51 (31%)	No or low activity	31/35 (89%)
Smoking	0/51 (0%)	Moderate to high activity	4/35 (11%)
Alcoholism	3/51 (5.9%)	Immunological biomarkers	
Heart disease	4/51 (7.8%)	Antinuclear Factor (ANA) positive	33/35 (94%)
Diabetes mellitus	3/51 (5.9%)	Rheumatoid Factor positive	17/38 (45%)
Chronic renal disease	1/51 (2.0%)	Anti-Ro positive	25/44 (57%)
Systemic arterial hypertension	7/51 (14%)	Anti-La positive	9/32 (28%)
Previous history of COVID-19	16/51 (31%)	C3 low	3/40 (7.50%)
		C4 low	2/39 (5.13%)

^1^ Mean ± SD; n/N (%); BMI: Body Mass Index; DMARDs: Disease-Modifying Antioxidant Drugs; ESSDAI: Sjogren’s Disease Systemic Activity Index; ANA: Antinuclear factor.

**Table 2:** Vaccination schedules are applied in primary immunization and booster vaccination in the population with Sjögren’s disease.

Characteristic	N = 51 ^1^
Primary Vaccination	
ChAdOx1 nCoV-19	48/51 (94%)
Inactivated SARS-CoV2 vaccine	3/51 (5.9%)
Schedule type	
Heterologous	37/51 (73%)
Homologous	14/51 (27%)
Vaccination schedule	
ChAdOx1 nCoV-19 + ChAdOx1 nCoV-19 + BNT162b2	34/51 (67%)
ChAdOx1 nCoV-19 + ChAdOx1 nCoV-19 + ChAdOx1 nCoV-19	14/51 (27%)
inactivated + inactivated + BNT162b2	3/51 (5.9%)

^1^ n/N (%).

**Table 3:** Simple Regression Evaluating the Association of Factors Associated with Humoral Response After COVID-19 Vaccination in Patients with Sjögren’s Disease.

Characteristic	N	Beta	95% CI ^1^	*p*-Value
Gender	51			
Male		—	—	
Female		−0.43	−1.9, 1.1	0.6
Age	51	0.02	−0.02, 0.06	0.3
Comorbidity	51	−0.45	−1.4, 0.46	0.3
COVID infection until T4	51	0.19	−2.1, 2.5	0.9
Pre-exposure to COVID	51	0.48	−0.44, 1.4	0.3
First dose	51			
Inactivated		—	—	
ChAdOx1		−0.89	−2.8, 1.0	0.4
Booster dose	51			
ChAdOx		—	—	
BNT162b2		1.9	1.0, 2.7	<0.001
Immunobiologicals	51	−1.3	−2.8, 0.16	0.087
Corticosteroid	51	0.77	−1.5, 3.1	0.5
Non-Immunosuppressed DMARDs or medication-free	51	0.08	−1.1, 1.2	0.9
DMARDs	51	−0.19	−1.3, 0.94	0.7
Immunosuppressants drugs	51	−0.23	−1.4, 0.95	0.7

^1^ CI = Confidence Interval. Note: The dash (—) indicates the reference (baseline) category used in the simple regression model.

**Table 4:** Local and systemic reactions after primary vaccination with Inactivated or ChAdOx1 and booster vaccination with ChAdOx1 or BNT162b.

	Primary Vaccination			Booster		
Characteristic	Inactivated N = 3 ^1^	ChAdOx1 N = 48 ^1^	*p*-Value ^2^	ChAdOx1 N = 14 ^1^	BNT162b2 N = 37 ^1^	*p*-Value ^2^
Local reactions	3/3 (100%)	44/48 (92%)	>0.9	10/14 (71%)	27/33 (82%)	0.5
Systemic reactions	1/3 (33%)	44/48 (92%)	0.033	10/14 (71%)	27/33 (82%)	0.5

^1^ n/N (%); ^2^ Fisher’s exact test.

**Table 5:** Reactions after primary vaccination schedule with inactivated vaccine or ChAdOx1 detailed by symptom.

Characteristic	Inactivated (N = 3 ^1^)	ChAdOx1 (N = 48 ^1^)	*p*-Value ^2^
Erythema	0/3 (0%)	12/48 (25%)	>0.9
Ecchymosis	0/3 (0%)	5/48 (10%)	>0.9
Lesion	1/3 (33%)	8/48 (17%)	0.4
Itching	0/1 (0%)	4/8 (50%)	>0.9
Swelling	0/3 (0%)	21/48 (44%)	0.3
Induration	0/3 (0%)	17/48 (35%)	0.5
Local pain	2/3 (67%)	43/48 (90%)	0.3
Nausea/Vomiting	1/3 (33%)	23/48 (48%)	>0.9
Tiredness	1/3 (33%)	35/48 (73%)	0.2
Headache	1/3 (33%)	35/48 (73%)	0.2
Muscle pain	1/3 (33%)	33/48 (69%)	0.3
Joint pain	1/3 (33%)	35/48 (73%)	0.2
Fever	0/3 (0%)	19/48 (40%)	0.3
Dizziness	0/3 (0%)	17/48 (35%)	0.5
Other complaints	0/3 (0%)	21/48 (44%)	0.3

^1^ n/N (%); ^2^ Fisher’s exact test.

**Table 6:** Reactions after booster with ChAdOx1 or BNT162b2 detailed by symptom.

Characteristic	ChAdOx (N = 14 ^1^)	BNT162b2 (N = 37 ^1^)	*p*-Value ^2^
Local reactions	10/14 (71%)	27/33 (82%)	0.5
Erythema	5/14 (36%)	11/33 (33%)	>0.9
Ecchymosis	3/14 (21%)	9/33 (27%)	>0.9
Lesion	2/14 (14%)	3/33 (9.1%)	0.6
Itching	0/2 (0%)	2/3 (67%)	0.4
Swelling	4/14 (29%)	17/33 (52%)	0.15
Induration	5/14 (36%)	15/33 (45%)	0.5
Local pain	10/14 (71%)	26/33 (79%)	0.7
Nausea/Vomiting	2/14 (14%)	5/33 (15%)	>0.9
Tiredness	7/14 (50%)	16/33 (48%)	>0.9
Headache	8/14 (57%)	18/33 (55%)	0.9
Muscle pain	6/14 (43%)	20/33 (61%)	0.3
Joint pain	7/14 (50%)	17/33 (52%)	>0.9
Fever	2/14 (14%)	5/33 (15%)	>0.9
Dizziness	3/14 (21%)	8/33 (24%)	>0.9
Other complaints	0/3 (0%)	21/48 (44%)	0.3

^1^ n/N (%); ^2^ Fisher’s exact test.

## Data Availability

The data are not publicly available due to privacy restrictions and are only available upon request to the corresponding author at val.valim@gmail.com.
